# The Effects of Different Varieties of *Aurantii Fructus Immaturus* on the Potential Toxicity of Zhi-Zi-Hou-Po Decoction Based on Spectrum-Toxicity Correlation Analysis

**DOI:** 10.3390/molecules24234254

**Published:** 2019-11-22

**Authors:** Qianqian Zhang, Fang Feng

**Affiliations:** 1 Department of Pharmaceutical Analysis, China Pharmaceutical University, Nanjing 210009, China; qianqianzhang618@163.com; 2 Key Laboratory of Drug Quality Control and Pharmacovigilance, Ministry of Education, China Pharmaceutical University, Nanjing 210009, China

**Keywords:** Zhi-Zi-Hou-Po decoction, spectrum–toxicity correlation analysis, synergistic effect, multivariate statistical analysis, potential toxicity

## Abstract

In accordance with the provision in *China Pharmacopoeia*, *Citrus aurantium* L. (Sour orange—SZS) and *Citrus sinensis* Osbeck (Sweet orange—TZS) are all in line with the requirements of *Aurantii Fructus Immaturus* (ZS). Both kinds of ZS are also marketed in the market. With the frequent occurrence of depression, Zhi-Zi-Hou-Po decoction (ZZHPD) has attracted wide attention. Currently, studies have shown that ZZHPD has a potential toxicity risk, but the effect of two commercial varieties of ZS on ZZHPD has not been reported. In this study, the toxicity differences of ZZHPD prepared by SZS and TZS were revealed through repeated administration experiments in rats. This indicated that different varieties of ZS could affect the toxicity of the prescription. In order to further study the chemical material basis of the toxicity difference, the fingerprints of ZZHPD prepared by different varieties of ZS were established by high-performance liquid chromatography (HPLC). Five different characteristic peaks were screened by non-target chemometrics. They were identified as geniposide, neoeriocitrin, naringin, hesperidin, and neohesperidin using an HPLC-time-of-flight mass spectrometry analyzer (TOF/MS) and an HPLC-triple stage quadrupole mass spectrometry analyzer (QqQ-MS/MS). Combined with a quantitative analysis and previous studies on promoting the intestinal absorption of geniposide, it is speculated that the synergistic effects of the components may be the main reason for the difference of toxicity among the different medicinal materials. This study provides a reference for the clinical, safe use of ZZHPD, and also provides a new perspective for the study of the potential toxic substances of traditional Chinese medicine compound preparations.

Academic Editor: Francesco Cacciola

## 1. Introduction

*Aurantii Fructus Immaturus* (ZS) is a commonly used traditional Chinese medicine with a long history of clinical application, and an excellent medicinal and edible value. It was first recorded in *Shen-Nong-Ben-Cao-Jing* (Sheng Nong’s herbal classic). Modern pharmacological studies have proven that it can promote gastric emptying, lower blood sugar and blood lipids, anti-oxidation, and lipid metabolism [1,2,3,4]. The 2015 edition of the *Chinese Pharmacopoeia* uses synephrine as an indicator to control the quality of ZS, and stipulates that ZS is the dried fruit and young fruit of *Citrus aurantium* L. (Sour orange—SZS) or *Citrus sinensis* Osbeck (Sweet orange—TZS). Therefore, SZS and TZS are both marketed as ZS in the market. Studies have, however, demonstrated that there are some differences in chemical composition and content between the two varieties of ZS [5,6]. Compared with SZS, some flavonoids in TZS are lower in content, which are important functional ingredients in ZS. A diversified chemical composition is the material basis for the efficacy and toxicity of traditional Chinese medicine compound preparations. However, there are currently few reports on whether there are differences in the clinical application of these two varieties of ZS.

With the increasing global incidence, depression has become one of the most important diseases affecting human mental health and quality of life. Although chemotherapy is effective in the treatment of depression, it has the disadvantages of withdrawal effects and recurrence [7]. Owing to the characteristics of multi-target, multi-channel, and multi-level interactions, searching for anti-depressant active ingredients from traditional Chinese medicine has become a hot research topic at home and abroad. Zhi-Zi-Hou-Po decoction (ZZHPD), which is made of *Gradenia jasminoides* Eills (ZZ), *Magnolia officinalis* cortex (HP), and ZS, has been used for many years as a representative prescription for the treatment of depression and related diseases [8]. Therefore, ZZHPD has attracted widespread attention for its efficacy and long use history. It is well known that the treatment of depression is a process that requires long-term medication. The possibility of hepatotoxicity after the repeated administration of ZZHPD has been confirmed by our laboratory, which is mainly related to gardenoside [9]. ZS is an important herbal medicine in ZZHPD, which has a synergistic effect, along with two other herbs. Although ZS is a non-toxic medicinal material, it is unknown whether the difference in its variety will affect the potential toxicity of ZZHPD. This uncertainty seriously affects the safety of the clinical application of ZZHPD.

Because the complex system of traditional Chinese medicine is synergistically exerted by multi-component, multi-level, and multi-dimensional effects, the difference in components may produce significant differences in efficacy or toxicity [10,11]. After the compatibility of the compound preparations, a physical or chemical reaction between the chemical components may be expected to result in a change in the type or content of the components, which ultimately changes the course of the drug in the body [12]. Therefore, the efficacy or toxicity of the traditional Chinese medicine compound preparation is not the result of a single medicine or a single ingredient, but a synergistic effect of various chemical components. Fingerprint and chemometric identification patterns are important methods for evaluating the overall quality of traditional Chinese medicine. Fingerprint identification can fully and comprehensively characterize the known and unknown components of traditional Chinese medicine, while chemometrics can digitally express, identify, and process the information of the fingerprint [13,14]. The combination of the two methods can reflect the quality information of Chinese medicinal materials more scientifically, objectively, and systematically. In combination with the correlation between the chemical constituents and its efficacy or toxicity, this method has been extensively used in the quality control, efficacy, and toxicity research of Chinese herbal medicines in recent years [15,16,17,18].

TZS and SZS have significant differences in their components, but they are both marketed as ZS. Whether the two kinds of ZS will affect the potential toxicity of ZZHPD is unknown. This poses a huge hidden danger to the safety of the clinical use of ZZHPD, with a potential toxicity. In response to this situation, we propose a comprehensive analysis strategy based on spectrum–toxicity correlation to analyze the effects of different varieties of ZS on the potential toxicity of ZZHPD. The first step of this study demonstrated the difference in toxicity after the repeated administration of ZZHPD containing different varieties of ZS. Then, it skillfully applied non-target chemometric processing methods to screen the key components, which led to different potential toxicities of ZZHPD with different ZSs through different characteristic peaks of the fingerprint. Furthermore, we discussed the synergistic effects of different components on the potential toxicity from a new perspective.

## 2. Results

### 2.1. Verification of Toxicity Difference

**Figure 1:**
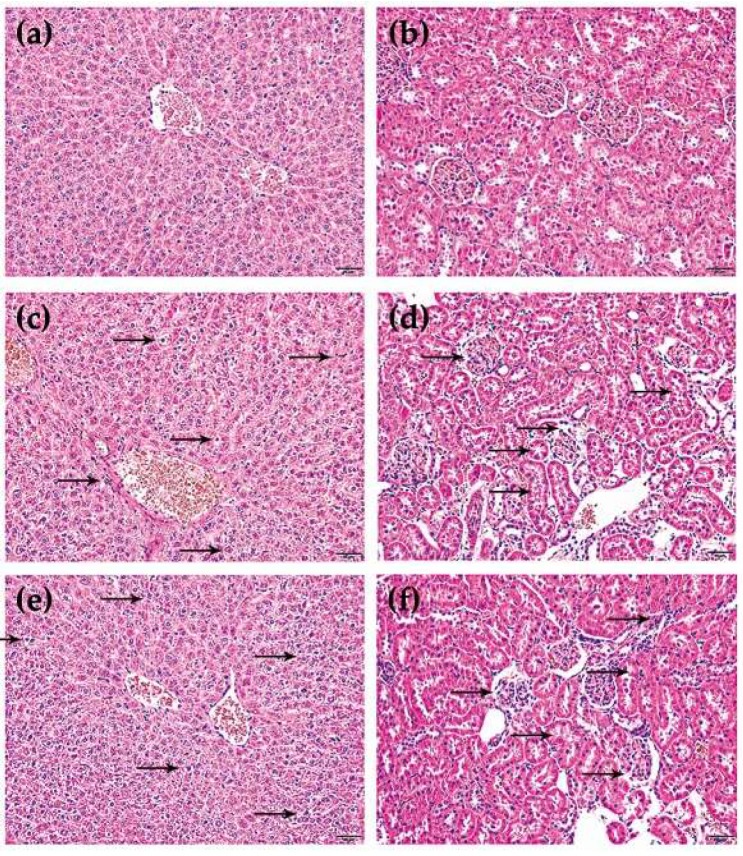
Typical histopathological section photographs of the rat liver and kidney specimens for H&E analysis (200 × magnifications). (**a**,**b**) are normal rat liver and kidney sections, (**c**,**d**) are the sections of liver and kidney of the Zhi-Zi-Hou-Po decoction (ZZHPD) prepared using *Citrus sinensis* Osbeck (Sweet orange—TZS) group, (**e**,**f**) are the sections of liver and kidney of the ZZHPD prepared using *Citrus aurantium* L. (Sour orange—SZS) group, respectively. The arrows show obvious lesion areas.

After five days of administration, the rats in the ZZHPD-dosed group began to be subjected to mental depression, lethargy, loose stool, and hair and perianal contamination. The control group had no obvious diarrhea or dirty fur, and was in a good mental state. Three rats in the ZZHPD with SZS group died after 11 and 12 days of administration, and the last two died after 16 days of administration. Two rats in the ZZHPD with TZS group died after 23 days of administration, and the remaining three rats died after about 25 days of administration. After autopsy, the liver and kidney of the control group were soft and ruddy. HE staining showed an unmistakable structure of hepatic lobules, no hepatocyte swelling or necrosis, and a normal glomerular and tubular morphology (Figure 1a,b). On the contrary, the liver parenchyma of the rats in the ZZHPD-dosed group was slightly stiff and dark red, and there were bleeding spots on the surface of the liver. The kidney was slightly enlarged and dark black. Figure 1c,e shows marked swelling and pyknosis of hepatocytes, with some naked and binuclear cells. The hepatocytes had mild vacuolar degeneration and a small amount of inflammatory cells infiltration. In the renal tissue, as shown in Figure 1d,f, the proximal convoluted tubule epithelium displayed apparent edema, the lumen was markedly dilated, the brush border detached, and inflammatory cell infiltration was observed. In addition, some glomerular abnormalities were visible. The results of the histopathology suggested that the repeated administration of ZZHPD caused damage to the liver and kidney of rats, but there was no significant difference between the ZZHPD prepared with SZS and TZS groups. Alanine aminotransferase (ALT) and aspartate aminotransferase (AST) are important indicators of liver function damage. Creatinine (CRE) and urea nitrogen (BUN) can reflect the health status of kidneys. As shown in Table S4, the serum biochemical indicators showed that, compared with the control group, the levels of ALT, AST, CRE, and BUN in the ZZHPD-dosed group increased in varying degrees. There was a tendency in the following order: SZS group > TZS group > control group. The results showed that ZZHPD caused damage to the liver and kidneys, and the damage of SZS group was more serious. Coupled with that, the survival time of the rats in the SZS group was shorter than that in the TZS group, indicating that the ZZHPD containing SZS was slightly more toxic.

### 2.2. Similarity Analysis by Chromatographic Fingerprint

**Figure 2:**
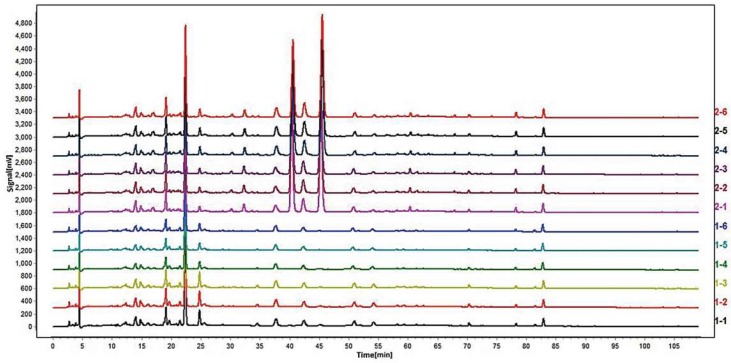
Fingerprint of ZZHPD prepared by TZS (1-1 to 1-6) and SZS (2-1 to 2-6).

In accordance with the preparation method of ZZHPD under Section 4.2., six ZZHPD samples were prepared in parallel with SZS and TZS, separately. The samples were analyzed by HPLC, and 12 data were obtained. The ZZHPD prepared using TZS was labeled 1-1 to 1-6, and the ZZHPD containing SZS was labeled 2-1 to 2-6. Then, the Analytical Instrument Association (AIA) format of the 12 ZZHPD samples was imported into the “Similarity Evaluation System for Chromatographic Fingerprints of TCM” (Chinese Pharmacopeia Commission, version 2004A) to evaluate the overall similarity of two batches of ZZHPD. The similarity results are shown in Tables S1–3. The similarity between the six samples of each ZZHPD was greater than 0.9, indicating that the parallelism of the sample preparation and the stability of the system were satisfactory [19]. However, the similarity between the two kinds of ZZHPDs is quite different, indicating that there are significant differences between them. It can be seen in Figure 2 that the ZZHPD prepared with TZS is obviously lacking several peaks compared with SZS.

### 2.3. Screening of Differential Components

**Figure 3:**
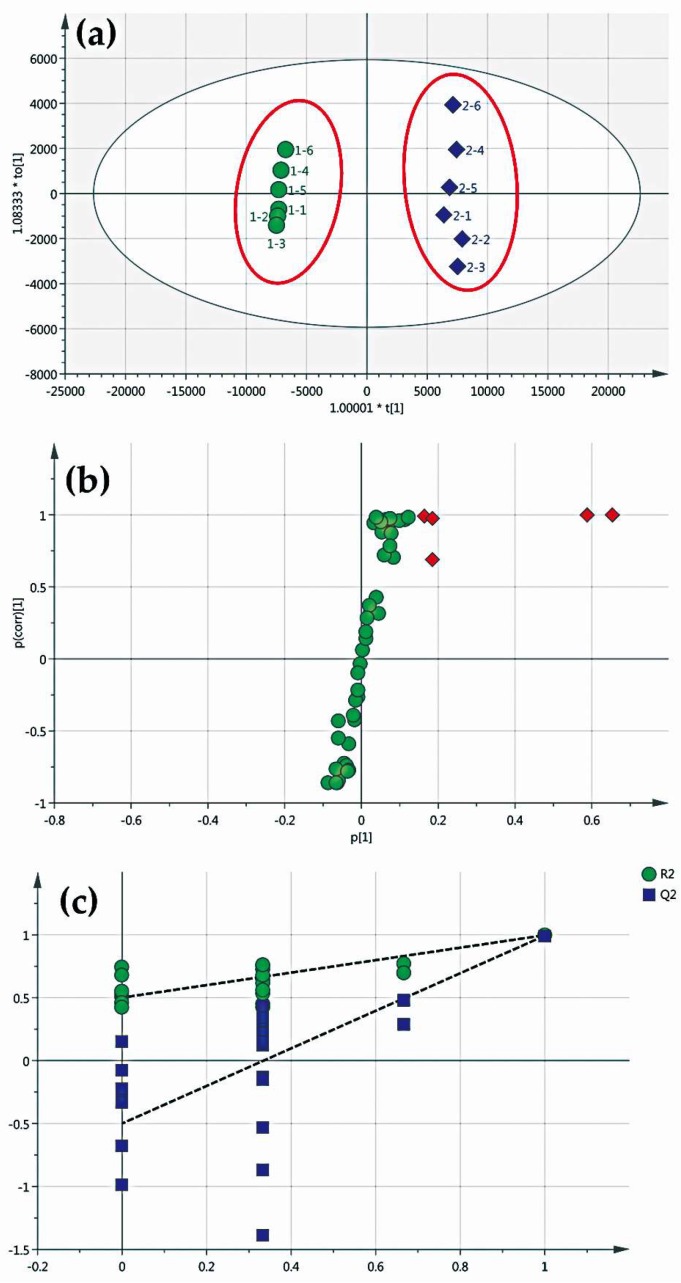
Multivariate statistical analysis of ZZHPD prepared using TZS and SZS. (**a**) OPLS-DA score plot (TZS is green dots and SZS is blue diamonds); (**b**) OPLS-DA S-plot (the variable importance in projection (VIP) value of the red diamonds are greater than 1); (**c**) model validation of the permutation tests.

**Figure 4:**
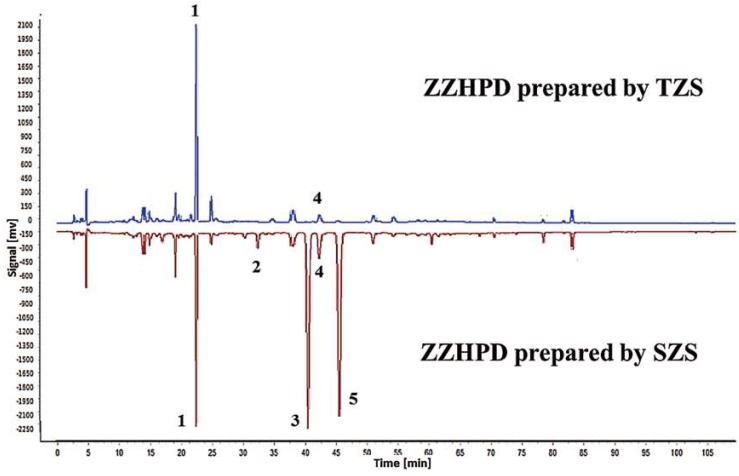
Image comparison of HPLC chromatograms between ZZHPD prepared using TZS and SZS (upper is TZS and lower is SZS); peaks 1–5 are the differential compounds identified.

In order to screen the different components of ZZHPD prepared using TZS and SZS, the common peak information identified by the fingerprint similarity software was imported into the SIMCA-P software for chemometric analysis. The variable importance in projection (VIP) value was calculated according to the orthogonal partial least squares discriminant (OPLS-DA) model (R^2^X = 0.901, R^2^Y = 0.997, Q^2^ = 0.993) so as to select the different peaks. In the OPLS-DA score plots (Figure 3a), the ZZHPD prepared with TZS (green dots) and SZS (blue diamonds) were obviously divided into two distinct groups, suggesting that there were potential differences in variables, leading to the dispersion of the two groups. So, the S-plot was used to visualize the variables that affected the model. Five differential variables (red diamonds), shown in Figure 3b, were screened out by the VIP value (>1), with retention times of 22.381, 32.293, 40.389, 42.310, and 45.319 min, respectively. The permutation test was used to verify the reliability of the model. As shown in Figure 3c, the intercept of Q^2^ on the Y-axis is less than zero, indicating that the model was reliable. In order to see the difference more intuitively, the HPLC chromatograms were compared in a mirror image (Figure 4). The labeled peaks are the significantly different components between the two kinds of ZZHPD samples.

### 2.4. Identification of Differential Component Peaks

**Figure 5:**
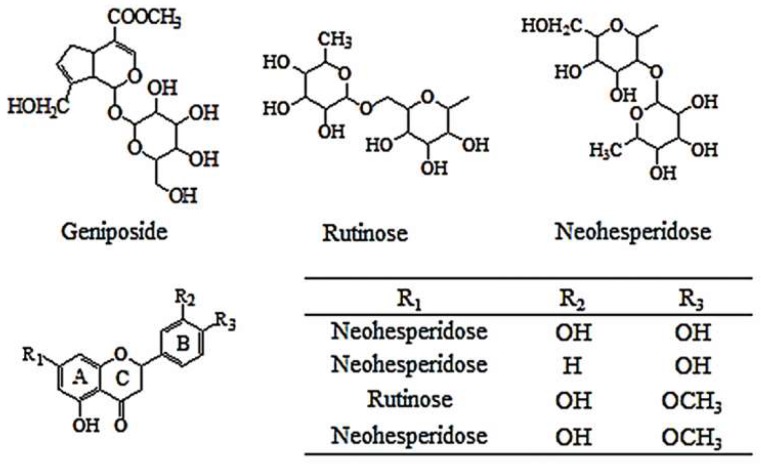
Chemical structures of differential compounds identified in different ZZHPDs.

In order to obtain the comprehensive characteristic of the differential compounds in ZZHPD, the screened differential peaks were analyzed using the LC–MS method under Section 4.5. The molecular formulas were calculated by high-accuracy quasi-molecular ions, such as [M + H]^+^, [M + Na]^+^, [M + CH_3_COO]^−^, and [M − H]^−^, within a mass error of 10 ppm. Then, the most reasonable molecular formula was searched in the previously reported ZZHPD compounds. The fragment ions obtained by HPLC-TOF/MS and/or HPLC-QqQ-MS/MS further confirmed the chemical structures of the compound, and ultraviolet (UV) absorption was also another important piece of evidence for speculating the type of compound. As a result, five compounds, namely geniposide, neoeriocitrin, naringin, hesperidin, and neohesperidin, were tentatively identified (Figure 5). The relevant identification information of the HPLC-TOF/MS and HPLC-QqQ-MS/MS in a positive or negative ion mode is listed in Table 1.

#### 2.4.1. Identification of Iridoid Glycosides

**Figure 6:**
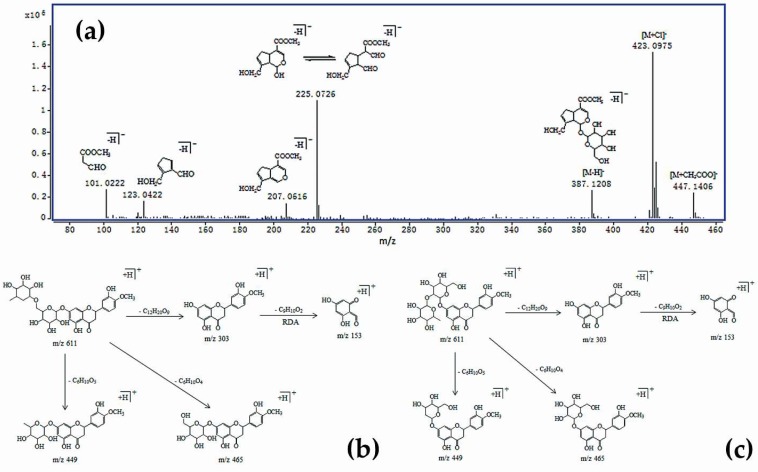
The HPLC-time-of-flight mass spectrometry analyzer (TOF/MS) spectra and proposed fragmentation analysis of geniposide, (**a**) and the detailed fragmentation pathways of hesperidin (**b**) and neohesperidin (**c**) in the positive ion mode.

Peak 1 has a maximum UV absorption at 240 nm, which is consistent with the UV absorption of the iridoid glycosides. In the full scan mode of HPLC-TOF/MS, the iridoid glycosides readily form [M + Na]^+^, [M + Cl]^−^, [M + CH_3_COO]^−^, and [M − H]^−^ molecular ion peaks. Under collision induced dissociation (CID) conditions, the [M − H]^−^ ions readily eliminated a glycosyl group to produce [Aglycone − H]^−^. As a result of the characteristic reaction of iridoid glycosides with a hemiacetal structure, the fragment ions at *m*/*z* 123 ([C_7_H_8_O_2_ − H]^−^) and *m*/*z* 101 ([C_4_H_6_O_3_ − H]^−^) were yielded by structural isomerization and bond breaking [20]. The molecular ion peak, [M − H]^−^ *m*/*z* 387, of peak 1 split and lost a neutral molecule, C_6_H_10_O_5_, and produced fragment ions of *m*/*z* 225. The fragment ion *m*/*z* 225 lost an H_2_O and produced fragment ions of *m*/*z* 207. As a result of the presence of a hemiacetal structure in *m*/*z* 225, the structural transformation caused the six-membered ring to open so as to form functional isomers. The functional isomer further lost a neutral molecule, C_7_H_8_O_6_, to form *m*/*z* 101, or lost a C_4_H_6_O_3_ to form *m*/*z* 123 through a retro-Diels–Alder (RDA) reaction. Combined with the literature [20], it is concluded that peak 1 is geniposide, and Figure 6a is the HPLC-TOFMS mass spectra and proposed fragmentation pathway of geniposide.

#### 2.4.2. Identification of Flavonoids

The flavonoid compounds have maximum absorption at 270–295 nm, while peaks 2, 3, 4, and 5 have a maximum UV absorption at 284 nm. It can be inferred that these peaks may be flavonoid compounds. In the HPLC-TOF/MS analysis, the glycosidic bonds of the O-glycosides in the flavonoids were easily cleaved to form 2-phenylchromone. The RDA reaction then readily occured on the C-ring of the flavonoids to produce corresponding ions. When the phenolic hydroxyl group and ketone group are attached to the parent nucleus structure, B-ring cleavage and CO loss will occur. However, mass spectrometry is difficult to distinguish the flavonoids with the same aglycone and glycosyl groups, but different glucose binding sites [21]. The difference in the binding sites will affect the polarity of the compound to some extent, resulting in different retention timed during the elution process. Therefore, such compounds can be inferred by being compared with the elution order in the literature.

In the positive ion mode, peak 2 produced molecular ion peaks of [M + Na]^+^ *m*/*z* 619 and [M + H]^+^ *m*/*z* 597. Its fragment ion were inferred to be [M + H − C_6_H_10_O_4_]^−^ *m*/*z* 451, [M + H − C_6_H_10_O_5_]^−^ *m*/*z* 435, and [M + H − C_12_H_20_O_9_]^−^ *m*/*z* 289, while *m*/*z* 289 further loses a C_8_H_8_O_2_ to form *m*/*z* 153, because of the RDA reaction. As the cleavage pathway is consistent with that reported in the literature [22], it can be inferred that peak 2 is neoeriocitrin. Meanwhile, peak 3, with [M + Na]^+^ *m*/*z* 603 and [M + H]^+^ *m*/*z* 581, was deduced to be naringin in the similar way.

Both peaks 4 and 5 produced molecular ion peaks of [M + H]^+^ *m*/*z* 611, which should be a group of isomers. As seen in Figure 6b,c, [M + H]^+^ *m*/*z* 611 loses a neutral molecule, C_6_H_10_O_4_, to generate *m*/*z* 465, or loses a C_6_H_10_O_5_ to form *m*/*z* 449. When it loses a C_12_H_20_O_9_ neutral molecule to form *m*/*z* 303, *m*/*z* 303 will further lose a C_9_H_10_O_2_ to form *m*/*z* 153, because of the RDA reaction. Its cleavage pathway is consistent with the reported hesperidin and neohesperidin pathways [21]. As the retention time of hesperidin is shorter in reversed-phase chromatography, peaks 4 and 5 are recognized as hesperidin and neohesperidin, respectively.

## 3. Discussion

According to the literature, geniposide is the main potential toxic component of ZZHPD. The long-term or large-scale administration of ZZ will cause a certain degree of damage to the liver and kidneys of rats [23]. The toxicity of ZS and HP has rarely been reported. In this study, the main difference between the two kinds of ZZHPDs lies in the different chemical components of different cultivars of ZS, which leads to some differences in toxicity. We speculate that there may be three reasons for the differences in the toxicity of ZZHPD, caused by different cultivars of ZS. The first point is the difference in chemical composition, resulting in the different dissolution of components during co-decoction. Secondly, the synergistic effect of different components has an effect on the absorption of potentially toxic components. Moreover, the inhibitory effect of differential components on CYP450 results in the accumulation of toxic components in tissues, which leads to an aggravation of toxicity.

Through the chemometric analysis and LC–MS/MS identification, five main different components affecting the two kinds of ZZHPD were screened out. In order to further study the differences in content, we used the external standard method to carry out a simple quantitative analysis of these differential components. The results of the composition content are shown in Table 2. In the ZZHPD prepared by TZS, neoeriocitrin, naringin, and neohesperidin did not meet the limit of quantitation. The results were consistent with the literature [6]. Meanwhile, it can be seen that the content of geniposide in the ZZHPD decocted with SZS is slightly higher than that of TZS. The content of flavonoids is different from that of the different cultivars of ZS, but the content of geniposide from the same ZZ is also different in the two kinds of ZZHPD. This indicated that the different components of ZS could affect the dissolution of geniposide, which is the main potential toxic component. The high content of neoeriocitrin, naringin, hesperidin, and neohesperidin in SZS may cause the dissolution of geniposide to be large, which makes the toxicity of ZZHPD more obvious.

On the other hand, our research team used the one-way perfusion method to evaluate the effect of the compatibility of ZZHPD on the intestinal absorption of geniposide. By establishing a one-way intestinal perfusion model in rats, the concentration of geniposide in the rat intestinal perfusate was determined by HPLC, and the absorption parameters of geniposide in different compatibility groups were calculated [24]. The results showed that the absorption rate constant (Ka) and effective permeability coefficient (Peff) of ZZ-SZS was significantly higher than those of the ZZ decoction (*p* < 0.05). In other words, the flavonoids in SZS can promote the intestinal absorption of geniposide. It has been reported that the absorption mechanism of flavonoid glycosides, such as naringin, hesperidin, and neohesperidin, in the human body is through the metabolism of intestinal flora to produce flavonoid aglycones, and is absorbed into the blood through the intestinal tract [25,26]. Geniposide has a similar absorption mechanism [27], so it is speculated that the combination of ZS and ZZ will compete for intestinal flora metabolism, thereby increasing the local intestinal concentration of geniposide and increasing absorption. Therefore, the difference in flavonoids in the different varieties of ZS can alter the pharmacokinetics of geniposide, leading to an increase in systemic exposure. It further affects the potential toxicity of ZZHPD.

Previous studies in this group have found that the accumulation of geniposide in the liver tissue may be related to the liver injury. CYP450 isoenzymes are mainly in the liver and extra hepatic tissues, which are responsible for about 90% of drug oxidation metabolism. The inhibition of CYP450 may induce toxicity by enhancing the exposure of the affected drugs. The flavonoid glycoside components in the SZS, such as hesperidin, naringin, and neohesperidin, have relatively strong inhibitory effects on CYP3A4 [28,29]. By inhibiting the activity of the CYP450 enzyme, it affects the metabolism of geniposide, causing its accumulation in the liver to result in a toxicity reaction. As the content of flavonoids in TZS is significantly lower than that of SZS, its inhibitory effect on the CYP450 enzyme is relatively weak, which reduces the accumulation of geniposide in the liver tissue, and shows a less toxic phenomenon.

In summary, the toxicity of ZZHPD is not a simple addition of the corresponding effects of a single herb, but a result of the synergistic and comprehensive effect of prescriptions. Because of the different chemical compositions, different varieties of herbs will change the overall effect of the prescription. However, in the *Chinese Pharmacopoeia*, the identification and quality control of ZS is based on synephrine and its content. The main difference between the two kinds of ZS is that the SZS contains high content of neoeriocitrin, naringin, hesperidin and neohesperidin, while the TZS contains almost no naringin and neohesperidin. Therefore, the SZS and TZS all meet the quality requirements of the *Pharmacopoeia*, and commercial sales include two varieties. It has led to potential safety hazards in the clinical use of traditional Chinese medicine compound preparations. This suggests that the quality control of traditional Chinese medicine should not only be carried out by a single component, but also by the synergistic effect of a multi-component and multi-target. A synergistic effect should be emphasized in the basic research on the efficacy and toxicity of traditional Chinese medicine compound preparations.

## 4. Materials and Methods

### 4.1. Chemicals and Materials

ZZ (no. 1702046; Jiangxi) and HP (no. 180404; Sichuan) were purchased from LBX Pharmacy Chain Co., Ltd. (Nanjing, China). ZSs of different varieties, produced in Jiangxi Province, China, were purchased from different Chinese herbal medicine markets located in Nanjing, Jiangsu Province. Among which, the batch number of SZS was no. 20170401 and TZS was no. 160928. In addition, they were authenticated by Professor Minjian Qin (Department of Chinese Materia Medica, China Pharmaceutical University, Nanjing, China).

Reference standards of geniposide, naringin, hesperidin, and neohesperidin (purity >98%) were purchased from Mansite Biotechnology Company (Chengdu, China).

HPLC-grade acetic acid and methanol were purchased from Sigma Chemical (St. Louis, MO, USA) and Merck (Darmstadt, Germany), respectively. Ultra-pure water was freshly obtained from a Milli-Q system (Millipore, Bedford, MA, USA). All of the other chemicals and reagents were analytical grade or higher, and commercially available.

The following programs were applied during the data processing: LC-Solution (Shimadzu Corp., Kyoto, Japan), SIMCA-P version 14.1 (Umetrics, Umea, Sweden), Mass Hunter B.04.00 (Agilent Corp., Santa Clara, CA, USA), Xcalibur 4.1 (Thermo fisher Scientific Waltham, MA, USA), and Microsoft Excel 2010 (Microsoft Corp., Redmond, Washington, USA).

### 4.2. Preparation of ZZHPD Sample

The ZZHPD samples were determined according to the clinical composition of depression treatment, and were prepared on the basis of a mature method developed in our laboratory. All three crude drugs were ground into powder before use. The weight of ZZ was 20 g, HP 15 g, and ZS 12 g, and they were soak for half an hour in the water (1:10, *w*/*v*). Then, they were boiled and simmered for half an hour on a low heat, and filtered with six layers of gauze. The process was repeated twice, with a ratio of the total herbal weight to water volume of 1:8 and 1:5, respectively. The three extracts were combined and concentrated, and then the solution was freeze-dried and stored at −20 °C before use. We dissolved an appropriate amount of freeze-dried powders with water before oral administration.

### 4.3. Animal Treatments and Sample Collection

All of the protocols and care of the rats were in accordance with the Guidelines for the Care and Use of Laboratory Animals, and approved by the Animal Ethics Committee of China Pharmaceutical University (SYXK (Su) 2018-0019). Male Sprague-Dawley rats (6–9 weeks) weighing 180–220 g were provided by the Animal Multiplication Center of Qinglong Mountain (Nanjing, China, SCX (Su) 2017-0001). Before oral treatment, the rats were acclimated in an animal breeding room for 7 days, with a temperature of 20 ± 2 °C, relative humidity of 40–50%, and light/dark cycle of 12 h. They were allowed free access to pure water and standard food.

Fifteen rats (no. 201828974) were randomly divided into the control group, SZS group, and TZS group (*n* = 5). All of the rats were fasted with free access to water for 12 h prior to the experiment. Different kinds of ZZHPDs containing SZT and TZS were orally administrated to the treatment group at a dose of 23.42 g/kg/d (equivalent to five times that of the clinical dosage) for four weeks, respectively, while an equal volume of distilled water was orally administrated to the control group.

### 4.4. Serum Biochemistry Assay and Histopathology

The blood samples were collected from the femoral vein in a tube without an anticoagulant. The whole blood of the rats was centrifuged at 1744× *g* for 15 min at 4 °C, so as to obtain the serum samples. The ALT, AST, CRE, and BUN levels were determined according to the manufacturer’s instructions, using a biochemical analyzer. The liver and kidney tissues of rats were taken immediately for gross morphological observation. Then, we cleaned the remaining blood on the surface of the tissue with normal saline, and dried it with filter paper. After that, the liver and kidney samples were stored in a 10% formaldehyde solution and embedded in paraffin. The paraffin sections were dewaxed and rehydrated under different alcohol gradients. Subsequently, HE staining was performed so as to observe the histopathological changes using a standard optical microscope (Olympus BX53, Tokyo, Japan).

### 4.5. LC–MS Analysis

#### 4.5.1. Sample Preparation

First, 7.0 mL of 95% ethanol was added into a 2.5 mL decoction, which formed a 70% ethanol precipitation solution. The mixture was then stored at 4 °C for 24 h to precipitate the protein and polysaccharides. The upper layer was centrifuged at 17,000× *g* for 10 min and then filtered through a 0.45 μm membrane filter before introducing it to HPLC analysis. 10 μL aliquots of the supernatant were injected into the HPLC-TOF/MS system and HPLC-QqQ-MS/MS system.

Six reference standards, magnolol, honokiol, geniposide, naringin, hesperidin and neohesperidin, were dissolved with methanol to obtain stock solutions at approximately 1.0 mg/mL and stored at 4 °C. The solutions were diluted with methanol for working solutions.

#### 4.5.2. Method Development

The HPLC-MS analysis was performed on an Agilent-1260 LC system coupled with an Agilent-6224 time-of-flight mass spectrometer (Agilent Corp., Santa Clara, CA, USA) and a TSQ AM tandem mass spectrometer (Thermo Finnegan, San Jose, CA, USA), both equipped with an electrospray interface (ESI). A Lichrospher-C18 column (250 × 4.6 mm, 5 μm; Hanbon, China) kept the column temperature at 35 °C. The mobile phase, consisting of (A) methanol and (B) 0.1% acetic acid, was carried with a linear elution gradient as follows: 0–15 min, 10–30% A; 15–25 min, 30–36% A; 25–49 min, 36–42% A; 49–66.5 min, 42–70% A; 66.5–86.5min, 70–81% A; 86.5–96.5 min, 81–95% A; and 96.5–109 min, 95% A. The flow rate was 0.9 mL/min. The re-equilibration duration was 10 min between the individual runs. The injection volume was 10 μL.

The HPLC-TOF/MS analysis was performed in both positive and negative ion modes in the range of 100–1000 *m*/*z*. The optimized conditions of the Electron Spray Ionization (ESI) source were a drying gas (N_2_) flow rate of 10.0 L/min, drying gas temperature of 350 °C, nebulizer pressure of 30 psig, fragmentor of +200/−135 V, and capillary voltage of +4.0/−3.5 kV. The wavelength range of the UV scan was set at 200.0–600.0 nm. The acquisition and analysis of the data were controlled by Mass Hunter B.04.00 software.

HPLC-QqQ-MS/MS was utilized to detect the product ion mass spectra in both the positive and negative ion modes. The optimized conditions of the ESI source were an ion spray voltage of +4.0/−3.5 kV, heated capillary temperature of 350 °C, sheath gas (N_2_) of 35 arbitrary, auxiliary gas (N_2_) of 5 arbitrary units, collision gas of 1.3 mTorr, collision energy of 8–35 eV, and a mass range of 50–1000 *m*/*z*. The spectral data were processed by Xcalibur 4.1 software.

#### 4.5.3. Method Validation

We ran the quality control sample six times before testing the sample, so as to adjust or balance the system. The method repeatability was evaluated by an analysis of six replicate of QC samples over a day. The stability of prepared sample was tested by running six prepared QC samples kept in auto sampler (maintained at 4 °C) for 12 h. In addition, one QC sample was analyzed for each of the six samples analyzed. Quality control data samples were collected to monitor the variability of the actual samples throughout the analysis. The relative standard deviations (RSDs) of the retention time (t_R_) and ion intensity of the components that were identified were calculated. The RSDs of t_R_ were below 2%, and the ion intensities were less than 10%. The results showed that the proposed method was satisfactory and suitable for the component analysis of ZZHPD.

#### 4.5.4. Data Analysis

The ion intensity of the peaks was used for the OPLS-DA analysis by SIMCA-P (version 14.1). The permutation test was used to verify the reliability of the model. The corresponding VIP value, calculated by the OPLS-DA model, was used to screen the characteristic components, followed by fingerprint analysis. Variables with a VIP >1 were considered to be differential components. In the identification of the differential components, the raw LC–MS data generated from the HPLC-TOF/MS and HPLC-QqQ-MS/MS analyses were processed using Mass Hunter B.04.00 software and Xcalibur 4.1 software.

## 5. Conclusions

Based on the multivariate statistical screening and HPLC-TOFMS and HPLC-QqQ-MS/MS identification, five different components of ZZHPD containing different varieties of ZS were analyzed. Through a correlation analysis of the spectrum–toxicity relationship, it is indicated that the potential toxicity of ZZHPD is related to the synergistic effect between the components. That is to say, the potential toxicity of the compound preparations is not only related to the toxic components, but also the result of the synergistic effects of various components. This study provides a reference for the quality control and clinical safe use of ZZHPD, and also provides a new idea for the study of potential toxic compounds of traditional Chinese medicine compound preparation.

## Figures and Tables

**Table 1:** The relevant identification information from both HPLC-TOF/MS and HPLC-QqQ-MS/MS, in a positive or negative ion mode.

No.	t_R_ (min)	HPLC-TOF/MS (*m*/*z*)	HPLC-QqQ-MS/MS		UV (nm)	Mass Weight	Formula	Identification	VIP
			+	−					
1	22.381	411.1253 [M + Na]^+^ 423.0975 [M + Cl]^−^ 447.1406 [M + CH_3_COO]^−^ 387.1208 [M − H]^−^ 775.2473 [2M − H]^−^	227, 209, 203	225, 207, 123, 101	240	388	C_17_H_24_O_10_	Geniposide	1.54751
2	32.293	619.1629 [M + Na]^+^ 597.1806 [M + H]^+^ 595.1662 [M − H]^−^	451, 435, 289, 153	449, 287, 151	284	596	C_27_H_32_O_15_	Neoeriocitrin	1.06007
3	40.389	603.1739 [M + Na]^+^ 581.1900 [M + H]^+^ 579.1750 [M − H]^−^	435, 419, 273, 153	459, 271, 151	283	580	C_27_H_32_O_14_	Naringin	4.06373
4	42.310	633.1789 [M + Na]^+^ 611.1964 [M + H]^+^ 609.1819 [M − H]^−^	465, 449, 303, 153	645, 301, 151	284	610	C_28_H_34_O_15_	Hesperidin	1.29501
5	45.319	633.1847 [M + Na]^+^ 611.0051 [M + H]^+^ 609.1861 [M − H]^−^	465, 449, 303, 153	609, 642, 151	284	610	C_28_H_34_O_15_	Neohesperidin	4.30420

**Table 2:** Results of the quantitative analysis of differential components.

ZZHPD	Geniposide (%)	Neoeriocitrin (%)	Naringin (%)	Hesperidin (%)	Neohesperidin (%)
TZS	3.70508	- ^1^	- ^1^	0.40404	- ^1^
SZS	3.88513	0.54788	9.23816	1.12065	7.98138

^1^ signifies that the component does not reach the limit of quantitation under this test condition.

## Supplementary Materials

The following are available online: Table S1: Results of similarity evaluation of ZZHPD prepared by TZS; Table S2: Results of similarity evaluation of ZZHPD prepared by SZS; Table S3: Results of similarity evaluation between different ZZHPD; Table S4: Serum biochemical indexes of different groups of rats.
