# Supplementary material for: The Effects of Different Varieties of Aurantii Fructus Immaturus on the Potential Toxicity of Zhi-Zi-Hou-Po Decoction Based on Spectrum-Toxicity Correlation Analysis
Source: Molecules. 2019 Nov 22;24(23):4254. doi: 10.3390/molecules24234254 (PMC6930465; doi:10.3390/molecules24234254)
Supplement: Supplementary file 1 [file molecules-24-04254-s001.pdf]

molecules

MDPI

Article

The Effects of Different Varieties of *Aurantii Fructus Immaturus* on the Potential Toxicity of Zhi-Zi-Hou-Po Decoction Based on Spectrum-Toxicity Correlation Analysis

Qianqian Zhang [1] and Fang Feng [1,2,*]

[1] Department of Pharmaceutical Analysis, China Pharmaceutical University, Nanjing 210009, China; qianqianzhang618@163.com
[2] Key Laboratory of Drug Quality Control and Pharmacovigilance, Ministry of Education, China Pharmaceutical University, Nanjing 210009, China
* Correspondence: fengfang1@hotmail.com; Tel.: +86-139-5168-2985

Supplementary Materials

Table S1. Results of similarity evaluation of ZZHPD Prepared by TZS.

No.	1-1	1-2	1-3	1-4	1-5	1-6	R
1-1	1	0.971	0.993	0.987	0.983	0.979	0.988
1-2	0.971	1	0.983	0.971	0.994	0.987	0.992
1-3	0.993	0.983	1	0.983	0.986	0.977	0.981
1-4	0.987	0.971	0.983	1	0.980	0.966	0.967
1-5	0.983	0.994	0.986	0.980	1	0.988	0.975
1-6	0.979	0.987	0.977	0.966	0.988	1	0.958
R	0.988	0.992	0.981	0.967	0.975	0.958	1

Table S2. Results of similarity evaluation of ZZHPD Prepared by SZS.

No.	2-1	2-2	2-3	2-4	2-5	2-6	R
2-1	1	0.987	0.982	0.947	0.954	0.944	0.977
2-2	0.987	1	0.968	0.980	0.967	0.948	0.971
2-3	0.982	0.968	1	0.974	0.959	0.964	0.978
2-4	0.947	0.980	0.974	1	0.953	0.942	0.973
2-5	0.954	0.967	0.959	0.953	1	0.958	0.961
2-6	0.944	0.948	0.964	0.942	0.958	1	0.954
R	0.977	0.971	0.978	0.973	0.961	0.954	1

Table S3. Results of similarity evaluation between different ZZHPD.

No.	1-1	1-2	1-3	1-4	1-5	1-6	2-1	2-2	2-3	2-4	2-5	2-6	R
1-1	1	0.991	0.983	0.987	0.982	0.976	0.382	0.375	0.356	0.386	0.377	0.371	0.641
1-2	0.991	1	0.987	0.979	0.963	0.959	0.378	0.388	0.367	0.381	0.369	0.364	0.627
1-3	0.983	0.987	1	0.937	0.944	0.958	0.392	0.373	0.361	0.357	0.365	0.374	0.648
1-4	0.987	0.979	0.937	1	0.951	0.963	0.374	0.369	0.350	0.376	0.366	0.348	0.633
1-5	0.982	0.963	0.944	0.951	1	0.972	0.379	0.365	0.351	0.388	0.360	0.362	0.624
1-6	0.976	0.959	0.958	0.963	0.972	1	0.386	0.379	0.367	0.391	0.359	0.347	0.651
2-1	0.382	0.378	0.392	0.374	0.379	0.386	1	0.979	0.981	0.988	0.992	0.987	0.952
2-2	0.375	0.388	0.373	0.369	0.365	0.379	0.979	1	0.973	0.990	0.981	0.974	0.967
2-3	0.356	0.367	0.361	0.350	0.351	0.367	0.981	0.973	1	0.963	0.975	0.944	0.935
2-4	0.386	0.381	0.357	0.376	0.388	0.391	0.988	0.990	0.963	1	0.969	0.962	0.952

2-5	0.377	0.369	0.365	0.366	0.360	0.359	0.992	0.981	0.975	0.969	1	0.981	0.944
2-6	0.371	0.364	0374	0.348	0.362	0.347	0.987	0.974	0.944	0.962	0.981	1	0.980
R	0.641	0.627	0.648	0.633	0.624	0.651	0.952	0.967	0.935	0.952	0.944	0.980	1

Table S4. Serum biochemical indexes of different groups of rats.

		**ALT(U/L)**	**AST(U/L)**	**CRE(μmol/L)**	**BUN(mmol/L)**
control group	1	55.2	118	28	6.02
	2	51.7	137	29	5.97
	3	46.5	138	25	5.94
	4	51.0	125	24	6.2
	5	48.8	142	25	5.68
	mean ± SD	50.64 ± 3.26	132.00 ± 10.07	26.20 ± 2.17	5.96 ± 0.19
SZS group	1	73	279	40	8.13
	2	--[1]	--[1]	--[1]	--[1]
	3	79.9	265	33	7.7
	4	83.4	296	39	7.88
	5	95.2	302	44	7.13
	mean ± SD	82.88 ± 9.28	285.50 ± 16.78	39.00 ± 4.55	7.71 ± 0.42
TZS group	1	69.8	283	31	6.91
	2	64.9	236	27	6.65
	3	66	227	31	7.8
	4	70.8	224	27	6.95
	5	--[1]	--[1]	--[1]	--[1]
	mean ± SD	67.88 ± 2.87	242.50 ± 27.48	29.00 ± 2.31	7.08 ± 0.50

[1] signifies the rat died in the middle of the night, its serum was not collected.
